# Accuracy of the clinical diagnosis of dementia with Lewy bodies (DLB) among the Italian Dementia Centers: a study by the Italian DLB study group (DLB-SINdem)

**DOI:** 10.1007/s10072-022-05987-z

**Published:** 2022-03-04

**Authors:** Mirella Russo, Claudia Carrarini, Angelo Di Iorio, Raffaello Pellegrino, Amalia Cecilia Bruni, Salvatore Caratozzolo, Annalisa Chiari, Stefano Pretta, Camillo Marra, Maria Sofia Cotelli, Andrea Arighi, Giorgio G. Fumagalli, Tatiana Cataruzza, Francesca Caso, Cristina Paci, Mara Rosso, Serena Amici, David Giannandrea, Andrea Pilotto, Simona Luzzi, Annalisa Castellano, Fabrizia D’antonio, Antonina Luca, Giorgio Gelosa, Tommaso Piccoli, Marco Mauri, Federica Agosta, Claudio Babiloni, Barbara Borroni, Marco Bozzali, Massimo Filippi, Daniela Galimberti, Roberto Monastero, Cristina Muscio, Lucilla Parnetti, Daniela Perani, Laura Serra, Vincenzo Silani, Pietro Tiraboschi, Annachiara Cagnin, Alessandro Padovani, Laura Bonanni, on Behalf of the Italian Dementia with Lewy bodies Study Group (DLB-SINdem), Baschi Roberta, Fragiacomo Federica, Galantucci Sebastiano, Gaspari Caterina, Gazzola Gianmarco, Magnani Giuseppe, Mazzon Giulia, Mozzetta Stefano, Ravanelli Carmela, Ruggiero Marco, Salute Pierpaolo, Scamarcia Pietro Giuseppe, Turla Marinella, Verde Federico, Volontè Maria Antonietta

**Affiliations:** 1 Department of Neuroscience, Imaging and Clinical Sciences, University G. d’Annunzio of Chieti-Pescara, Chieti, Italy; 2 Department of Medicine and Aging Sciences, University G. d’Annunzio of Chieti-Pescara, Chieti, Italy; 3 Regional Neurogenetic Centre, Department of Primary Care, ASP-CZ, Lamezia Terme, Italy; 4 Neurology Unit, Department of Clinical and Experimental Sciences, University of Brescia, Brescia, Italy; 5 U.O. Di Neurologia, Azienda Ospedaliera Universitaria Di Modena, Modena, Italy; 6 Neuroimaging Research Unit, Division of Neuroscience, IRCCS San Raffaele Scientific Institute, Milan, Italy; 7 Memory Clinic, Fondazione Policlinico Gemelli, IRCCS Università, Cattolica del Sacro Cuore, Rome, Italy; 8 UO Neurologia ASST Valcamonica, Brescia, Italy; 9 UOSD Neurologia, Malattie Neurodegenerative, Fondazione IRCCS Ca’ Granda Ospedale Maggiore Policlinico, Milano, Italy; 10 Department of Medicine, Surgery and Health Sciences, Neurology Unit, University Hospital and Health Services of Trieste, Trieste, Italy; 11 Unit of Neurology, IRCCS San Raffaele Scientific Institute, Milan, Italy; 12 Division of Neurology, Ospedale Madonna del Soccorso, ASUR Marche, San Benedetto del Tronto-Ascoli, Piceno Italy; 13 Neurology Clinic, SS Annunziata Hospital of Savigliano, Savigliano, Italy; 14 Cognitive Disorder and Dementia Unit, USL Umbria 1, Perugia, Italy; 15 Neurologia E Stroke Unit, Ospedale Di Gubbio E Gualdo Tadino, Perugia, Italy; 16 Department of Experimental and Clinical Medicine, Polytechnic University of Marche - Ospedali Riuniti, Ancona, Italy; 17 CDCD IRCSS Neuromed Di Pozzilli, Isernia, Italy; 18 Department of Human Neurosciences, Sapienza University of Rome, Rome, Italy; 19 Department of Medical, Surgical Sciences and Advanced Technologies, GF Ingrassia, University of Catania, Catania, Italy; 20 Cognitive Neuropsychology Center, Niguarda Hospital, Milan, Italy; 21 Unit of Neurology, Department of Biomedicine, Neurosciences and Advanced Diagnostics, University of Palermo, Palermo, Italy; 22 Department of Biotechnologies and Life Sciences, University of Insubria, Varese, Italy; 23 Unit of Neurology, Neuroimaging Research Unit, Division of Neuroscience, IRCCS San Raffaele Scientific Institute and Vita-Salute San Raffaele University, Milan, Italy; 24 Department of Physiology and Pharmacology “V. Erspamer, Sapienza University of Rome, Rome, Italy; 25 Hospital San Raffaele Cassino (FR), Cassino, Italy; 26 Department of Neuroscience, University of Turin, Turin, Italy; 27 Department of Biomedical, Surgical and Dental Sciences, University of Milan, Milan, Italy; 28 Section of Neurology, Department of Biomedicine, Neuroscience and Advanced Diagnostics, University of Palermo, Palermo, Italy; 29 Neurology 5-Neuropathology Unit, Fondazione IRCCS Istituto Neurologico Carlo Besta, Milan, Italy; 30 Laboratory of Clinical Neurochemistry, Section of Neurology, Department of Medicine and Surgery, University of Perugia, Perugia, Italy; 31 In Vivo Human Molecular and Structural Neuroimaging Unit, Division of Neuroscience, IRCCS San Raffaele Scientific Institute, Milan, Italy; 32 Neuroimaging Laboratory, Fondazione Santa Lucia, IRCCS, Rome, Italy; 33 Department of Neurology and Laboratory of Neuroscience, IRCCS Istituto Auxologico Italiano and Università Degli Studi Di Milano, Milano, Italy; 34 Department of Neurosciences, University of Padova, Padova, Italy

**Keywords:** Dementia with Lewy bodies, Diagnostic toolkits, Consensus criteria, Clinical diagnosis, Dementia, Cognitive impairment, Diagnostic accuracy

## Abstract

**Introduction:**

Dementia with Lewy bodies (DLB) may represent a diagnostic challenge, since its clinical picture overlaps with other dementia. Two toolkits have been developed to aid the clinician to diagnose DLB: the Lewy Body Composite Risk Score (LBCRS) and the Assessment Toolkit for DLB (AT-DLB). We aim to evaluate the reliability of these two questionnaires, and their ability to enhance the interpretation of the international consensus diagnostic criteria.

**Methods:**

LBCRS and AT-DLB were distributed to 135 Italian Neurological Centers for Cognitive Decline and Dementia (CDCDs), with the indication to administer them to all patients with dementia referred within the subsequent 3 months. We asked to subsequently apply consensus criteria for DLB diagnosis, to validate the diagnostic accuracy of the two toolkits.

**Results:**

A total of 23 Centers joined the study; 1854 patients were enrolled. We found a prevalence of possible or probable DLB of 13% each (26% total), according to the consensus criteria. LBCRS toolkit showed good reliability, with a Cronbach alpha of 0.77, stable even after removing variables from the construct. AT-DLB toolkit Cronbach alpha was 0.52 and, after the subtraction of the “cognitive fluctuation” criterion, was only 0.31. Accuracy, sensitivity, and specificity were higher for LBCRS vs. AT-DLB. However, when simultaneously considered in the logistic models, AT-DLB showed a better performance (*p* < 0.001). Overall, the concordance between LBCRS positive and AT-DLB possible/probable was of 78.02%

**Conclusions:**

In a clinical setting, the LBCRS and AT-DLB questionnaires have good accuracy for DLB diagnosis.

**Supplementary Information:**

The online version contains supplementary material available at 10.1007/s10072-022-05987-z.

## Introduction

Dementia with Lewy bodies (DLB), the second most common neurological cause of dementia after Alzheimer’s disease (AD), is characterized by cognitive fluctuations (CF), visual hallucinations (VH), parkinsonism, and REM sleep behavior disorder (RBD), which are considered as its core clinical features [1]. The accuracy of the clinical diagnosis of DLB is however not satisfactory, as the clinical presentation may overlap with AD [2].

The recent efforts of the researchers focusing on DLB have been addressed toward the identification of biomarkers which could help diagnose DLB as compared with AD.

Great emphasis has recently been placed on the necessity to identify more sensitive and specific diagnostic markers and to define the prodromal stage of DLB in order to put in place timely pharmacological and management interventions [3].

This research stream is witnessed by the recent flourishing of International Consortia on DLB (E-DLB, American Lewy Body Dementia (LBD) association, ISTAART LBD Professional Interest Area (PIA)). The efforts of the Consortia are aimed to overcome the challenges in recruiting sufficiently large and unbiased cohorts, and to identify which diagnostic instruments are sensitive to change in DLB.

Toward these aims, the Italian Neurological Society for dementia (SINdem) promoted the constitution of an Italian DLB study group. The general objectives of the study group were defined as follows:

- To improve DLB identification by physicians working in Centers for Cognitive Decline and Dementia (CDCDs).
- To identify the DLB cohorts available in Italy and develop an efficient method of data collection.

A first survey conceived to identify the DLB cohorts available in Italy was performed in 2016 [4]. The CDCDs were asked to answer a semi-structured questionnaire, which investigated the following: (1) incidence and prevalence of DLB; (2) clinical assessment; (3) relevance and availability of diagnostic tools; (4) pharmacological management of cognitive, motor, and behavioral disturbances; (5) causes of hospitalization, with specific focus on delirium and its treatment. Overall, 135 centers (23.6% of all CDCDs) contributed to that first survey. A total of 5624 patients with DLB were followed at the time of the survey by the 135 centers in a year (2042 of them were new referrals). The percentage of DLB patients among neurodegenerative dementia was 27 ± 8%.

The prevalence of DLB in the Italian dementia population appeared therefore to be far higher than the one reported in literature, which ranges from 5 to 15% [1].

We explained that result by judging a semi-structured questionnaire as insufficiently accurate to avoid catching up cases of mixed dementia, which could be wrongly classified as DLB, in a country where neurodegenerative disease cases are not neuropathologically confirmed.

The aim of the present study is to re-evaluate the prevalence of DLB patients by using two standardized questionnaires, based on clinical diagnostic criteria for DLB. The questionnaires were specifically designed to address the problem of inadequate recognition and diagnosis of DLB [5, 6].

The first questionnaire, the Lewy Body Composite Risk Score (LBCRS) [7], was designed to improve the ability to detect DLB in clinical and research populations and to increase the likelihood of determining whether Lewy bodies are a contributing pathology to the dementia syndrome. The LBCRS was derived from clinical features in autopsy-verified cases of healthy controls, AD, DLB, and Parkinson disease (PD) with and without dementia (PDD). The LBCRS was tested in comparison with gold standard measures of cognition, motor symptoms, function, and behavior.

The second questionnaire, the Assessment Toolkit for DLB (AT-DLB) [8], was developed to be aligned with the standard diagnostic criteria for DLB [1], and to be applied by clinicians in regular clinical services and easily integrated into routine care.

## Methods

In this cross-sectional observational study, the two questionnaires were distributed in March 2019 to the 135 neurological CDCDs, which participated in the previous survey. The CDCDs included were evenly distributed over the country from north to south. Out of the 23 participating Centers, 10 CDCDs (43%) are located within Neurology Units of University Hospitals, 5 CDCDs (22%) belong to Scientific Institute for Research, Hospitalization and Healthcare (IRCSS), 5 CDCDs (22%) are placed in Neurology Units of Non-Academic Hospitals, and 3 CDCDs (13%) belong to territorial out-patient clinics.

We asked to administer the surveys to all the patients with dementia (Mini-Mental State Examination (MMSE) score < 24) [9] referred to the Centers in the subsequent 3 months, independently from the initial suspected diagnosis and from the final diagnoses.

All Centers were trained to apply the most recent diagnostic criteria for DLB [1].

More specifically, the neurologists of each Center were instructed to carry out in each patient a physical and neurological examinations.

The presence of cognitive fluctuations was confirmed by detailed semi-structured interview and quantified using the Clinician Assessment of Fluctuations questionnaire [10]. Visual hallucinations were determined by detailed interview with the patient and caregiver followed by confirmation and quantification according to the Neuropsychiatric Inventory [11]. Parkinsonism was diagnosed by the Motor part (part III) of the Unified Parkinson’s Disease (PD) Rating Scale (UPDRS) [12]. Symptoms of RBD were assessed by interview and scored according to the Mayo Sleep Questionnaire [13]. The application of the DLB diagnostic criteria based on the aforementioned tests took about 60 min.

Each of the two questionnaires (LBCRS and AT-DLB) were administered to patients in random order.

The administration of the questionnaires took between 30 and 60 min, depending on the compliance of the patients and caregivers, and on the severity of the clinical picture. Considering the items included in each questionnaire, the aim was to compare the results of either LBCRS or AT-DLB with the recent criteria. Where considered clinically appropriate, the patients underwent neuroimaging and neurophysiology assessments, as DaT-SPECT, myocardial scintigraphy, FDG-PET for detection of the Cingulate Island Sign, Quantitative EEG, polysomnography to confirm RBD. All procedures were in accordance with the ethical standards of the institutional and national research committee and with the Helsinki Declaration. Informed consent was obtained by all participants.

### Lewy Body Composite Risk Score

The LBCRS [7] evaluates the presence of four motor and six non-motor symptoms within the last 6 months. Motor signs include slowness, rigidity, postural instability, and resting tremor. Non-motor symptoms are the following: excessive daytime sleepiness, illogical thoughts, frequent episodes of staring, visual hallucinations (VH), enacted dreaming, and autonomic dysfunctions.

The symptoms were considered as present if they occurred at least three times during the 6 months preceding the clinical investigation.

A global score equal or superior to 3 indicates a probable DLB diagnosis (LBCRS positive), whereas a score ranging from 0 to 2 is not suggestive of DLB diagnosis (LBCRS negative).

### Assessment Toolkit for Dementia with Lewy Bodies

The Assessment Toolkit for DLB [8] is based on a series of specific questions carried out to identify core and suggestive features of DLB. Beyond the evidence of global cognitive decline, four domains are investigated (core clinical features): CF, VH, RBD, and parkinsonism. The toolkit includes a questionnaire that is administered by the rater to the patient and the caregiver, and a short neurological exam to determine the 5-item Unified Parkinson’s Disease Rating Scale (UPDRS) score. Moreover, the presence of dopaminergic deficit in basal ganglia on SPECT/PET, low uptake on metaiodobenzylguanidine (MIBG) myocardial scintigraphy, or polysomnography confirmation of RBD is evaluated. These features are considered as indicative biomarkers. A diagnosis of probable DLB is made if either two core features or one core and one indicative feature are identified. A diagnosis of possible DLB is considered if one feature is satisfied.

### Statistical analysis

Statistical analysis was performed using Statistical Analysis Software (SAS).

Data were reported as mean ± standard errors, for continuous variables, and as absolute number and percentage for categorical ones.

To assess the differences in the prevalence of symptoms in the three study groups, logistic models were used, where the absence of DLB study group was the reference. Moreover, to test whether the magnitude of association of each risk factor with DLB diagnosis differed between the 2 DLB group (probable and possible), the equivalence of the odds ratios (ORs) was computed by a Mantel–Haenszel *χ*^2^ statistic based on the weighted sum of the squared deviations of the stratum-specific log ORs from their weighted mean [14].

The statistical agreement of LBCRS and AT-DLB with the most recent diagnostic criteria of DLB was calculated. To consider how each variable reflects the reliability of a scale with standardized variables, the standardized alpha coefficient (Cronbach alpha) was also estimated. If the standardized alpha decreases after removing a variable from the model, that variable is strongly correlated with other variables included in the scale and contributes to the reliability of the scale. Conversely, if the standardized alpha increases after removing a variable from the model, removing that variable makes the scale more reliable [15]. The rate of concordance between the DLB clinical criteria [16] and the two toolkits was evaluated by accuracy, sensitivity, and specificity calculation. Logistic regression models were applied to calculate receiver operating characteristics (ROC) and to estimate the area under the curve (AUC). In the models, DLB criteria were the dependent variable while LBCRS and AT-DLB were the independent variables, which were simultaneously considered. When ROC was estimated for the “possible DLB” group, those classified as “probable DLB” were excluded from the analysis, and vice versa.

## Results

Twenty-three CDCDs joined the research and performed the survey. Of the 2006 patients enrolled, 152 (7.58%) subjects were excluded because of missing data. Among the 1854 remaining patients, 1048 (56.53%) were female and the mean age of the sample was 75.06 ± 14.58 years. MMSE was 16.4 ± 7.1. All the patients underwent computed tomography/magnetic resonance imaging.

Seventy-four patients (4.7%) underwent DaT-SPECT, 5 patients (0.3%) underwent myocardial scintigraphy, 51 patients (2.8%) underwent FDG-PET for detection of the Cingulate Island Sign, 29 patients (1.6%) underwent quantitative EEG, and 5 patients (0.3%) underwent polysomnography to confirm RBD.

Our results showed a prevalence of possible or probable DLB of 13% each (26% total), according to the consensus criteria (Table 1).

**Table 1:** Patient diagnoses according to the consensus criteria for DLB (McKeith et al. 2017) at the two times of assessment

Patient diagnoses	Consensus criteria First assessment	Consensus criteria Second assessment
No DLB	73.46%	73.03%
Possible DLB	13.43%	13.75%
Probable DLB	13.11%	13.21%

Abbreviations: *DLB*, dementia with Lewy bodies

### Lewy Body Composite Risk Score

Applying the LBCRS in the study sample, 555 (30.66%) patients were classified as DLB (LBCRS positive). The Cronbach alpha showed good reliability of the scale (0.77). Indeed, removing variables from the model, the alpha coefficient ranged from 0.74 to 0.76.

Table 2 reports the frequencies of all the features included in the assessment, according to diagnosis reached using clinical DLB criteria. Among all items, those related to VH and RBD (questions 8 and 9) reported a higher ORs for diagnosis of probable DLB (VH-Q8: 40.61, 95%CI: 27.48–60.01; RBD-Q9: 31.31, 95%CI: 21.10–46.46).

**Table 2:** Frequencies of all features included in the LBCRS in comparison with diagnostic criteria

	No DLB	Possible DLB	OR (95%CI) No vs. possible	Probable DLB	OR (95%CI) No vs. probable	*p*-value
Patients (*n*)	1342	233		235		
Q1 Slowness	258 (19.2)	99 (42.5)	3.1 (2.3–4.2)	174 (74.0)	12.0 (8.7–16.5)	< 0.001
Q2 Rigidity	201 (15.0)	128 (54.9)	6.9 (5.1–9.3)	168 (71.5)	14.2 (10.3–19.6)	< 0.001
Q3 Postural instability	190 (14.2)	74 (31.8)	2.8 (2.1–3.9)	110 (46.8)	5.3 (4.0–7.2)	0.003
Q4 Rest tremor	57 (4.3)	29 (12.5)	3.2 (2.0–5.1)	67 (28.5)	9.0 (6.1–13.3)	0.004
Q5 Day sleepiness	159 (11.8)	54 (23.2)	2.3 (1.6–3.2)	126 (53.6)	8.6 (6.3–11.7)	< 0.001
Q6 Illogical thinking	287 (21.4)	72 (30.9)	1.6 (1.2–2.3)	122 (51.9)	4.0 (3.0–5.3)	< 0.001
Q7 Episodes of absence or staring off into space	231 (17.2)	77 (33.1)	1.8 (1.3–2.4)	116 (49.4)	4.0 (3.0–5.3)	< 0.001
Q8 Visual hallucination	47 (3.5)	54 (23.2)	8.3 (5.5–12.7)	140 (59.6)	40.6 (27.5–60.0)	< 0.001
Q9 Dreams enacted	44 (3.3)	44 (18.9)	6.9 (4.4–10.7)	121 (51.5)	31.3 (21.1–46.5)	< 0.001
Q10 Orthostatic hypotension	28 (2.1)	7 (3.0)	1.5 (0.6–3.7)	51 (21.7)	13.0 (8.0–21.2)	< 0.001

Data are reported as number of patients, *n* (%); *CI*, confidence interval; *OR*, odds ratio; *Q*, questions. The *p*-values* refer to test of comparison of the OR

### Assessment Toolkit for Dementia with Lewy Bodies

After performing AT-DLB, 445 (24.59%) patients were diagnosed as possible-DLB, whereas 322 (17.79%) were probable DLB. For AT-DLB, the Cronbach alpha was of 0.52. Only when removing the item “CF,” the alpha coefficient decreased to 0.31.

Table 3 shows the frequencies of symptoms, considered in this scale, according to the recent DLB criteria. Among all items, those related to CF and VH (questions 1 and 7–10) were associated with the five highest ORs for diagnosis of probable DLB (CF-Q1: 52.73, 95%CI: 36.06–77.09; RBD-Q7: 21.51, 95%CI: 13.43–34.47; RBD-Q8: 29.28, 95%CI: 18.93–45.29; RBD-Q9: 42.79, 95%CI: 28.13–64.18; RBD-Q10: 35.50, 95%CI: 24.20–52.07).

**Table 3:** Frequencies of all features included in AT-DLB in comparison with diagnostic criteria

	No DLB	Possible DLB	OR (95%CI) No DLB vs possible	Probable DLB	OR (95%CI) No DLB vs probable	*p*-value
Patients (*n*)	1342	233		235		
CF						
Q1 Changes in functioning	88 (6.6)	82 (35.2)	7.7 (5.5–10.9)	185 (78.7)	52.7 (36.1–77.1)	< 0.001
Q2 > 1 h day sleeping	82 (6.1)	50 (21.5)	4.2 (2.9–6.2)	86 (36.6)	8.9 (6.3–12.6)	< 0.001
Q3 > 1 h drowsy	305 (22.7)	141 (60.5)	5.2 (3.9–7.0)	159 (67.7)	7.1 (5.3–9.6)	0.05
Q4 Difficult to arouse	254 (18.9)	106 (45.5)	3.6 (2.7–4.8)	135 (57.5)	5.8 (4.3–7.8)	< 0.001
RBD						
Q5 Dream enact	56 (4.2)	15 (6.4)	1.6 (0.9–2.8)	106 (45.1)	18.9 (13.0–27.4)	< 0.001
Q6 Dream enact*	47 (3.5)	12 (5.2)	1.5 (0.8–2.9)	69 (29.4)	11.5 (7.6–17.2)	< 0.001
VH						
Q7 Eye tricks*	27 (2.0)	7 (3.0)	1.5 (0.7–3.5)	72 (30.6)	21.5 (13.4–34.5)	< 0.001
Q8 Sees things*	32 (2.4)	16 (6.9)	3.0 (1.6–5.6)	98 (41.7)	29.3 (18.9–45.3)	< 0.001
Q9 False visions	38 (2.8)	33 (14.2)	5.7 (3.5–9.2)	130 (55.3)	42.79 (28.13–64.18)	< 0.001
Q10 Sees things	50 (3.7)	32 (13.7)	4.1 (2.6–6.6)	136 (57.9)	35.50 (24.20–52.07)	< 0.001
Motor symptoms						
UPDRS 5-item score	0.8 ± 1.7	2.9 ± 2.7	3.4 (1.7–7.0)	4.82 ± 3.2	19.68 (11.64–33.28)	< 0.001

Unless marked by the *, which distinguishes questions posed to the patient, the questions are posed to the patient’s caregiver. Data are reported as number of patients, *n* (%). UPDRS 5-item score data are reported as mean ± standard deviation

Abbreviations: *CF*, cognitive fluctuations; *CI*, confidence interval; *OR*, odds ratio; *RBD*, REM sleep behavior disorder; *VH*, visual hallucinations; *Q*, questions. Data are reported as number of patients, *n* (%). The *p*-values* refer to test of comparison of the OR

### Comparison between Assessment Toolkit for Dementia with Lewy Bodies and Lewy Body Composite Risk Score

The percentage of agreement between AT-DLB possible and LBCRS positive was 32.43%, whereas the concordance between AT-DLB probable and LBCRS positive was higher (45.59%). Overall, the concordance between LBCRS positive and AT-DLB possible/probable was of 78.02% (see Table 4 for the comparison between the two assessments). The agreement between LBCRS negative and AT-DLB negative was 88.30%.

**Table 4:** Stratification of patients according to LBCRS and AT-DLB toolkits

	AT-DLB No DLB	AT-DLB Possible DLB	AT-DLB Probable DLB	Total	
LBCRS negative	921 50.88 **73.39** 88.30	265 14.64 21.12 59.55	69 3.81 5.50 21.43	1255 69.34	Frequency % (whole cohort) % (of the row) % (of the column)
LBCRS positive	122 6.74 21.98 11.70	180 9.94 **32.43** 40.45	253 13.98 **45.59** 78.57	555 30.66	Frequency % (whole cohort) % (of the row) % (of the column)
Total	1043 57.62	445 24.59	322 17.79	1810 100.00	

Bold text: percentage of patients, whose diagnosis with AT-DLB and LBCRS was concordant

Abbreviations: *DLB*, dementia with Lewy bodies; *AT-DLB*, Assessment Toolkit for DLB; *LBCRS*, Lewy Body Composite Risk Score; *LBCRS positive*, probable DLB according to LBCRS; *LBCRS negative*, subjects who did not meet the criteria for probable DLB according to the LBCRS toolkit

Table 5 shows the discrepancies between the two toolkits for attribution of patients to DLB diagnosis. Among all features, CF and VH showed a higher ORs for categorization of AT-DLB negative and LBCRS positive.

**Table 5:** Frequency of distribution for the items of AT-DLB, according to AT-DLB and LBCRS toolkits

	LBCRS negative AT-DLB positive	LBCRS positive AT-DLB positive	LBCRS negative AT-DLB negative	LBCRS positive AT-DLB negative	LBCRS positive AT-DLB positive vs LBCRS positive AT-DLB negative	LBCRS negative AT-DLB negative vs LBCRS negative AT-DLB positive
Patients (*n*)	334	433	921	122		
CF						
Q1 Changes in functioning	86 (25.75)	214 (49.42)	19 (2.06)	36 (29.51)	2.33 (1.52–3.60)	0.06 (0.04–0.10)
Q2 > 1 h day sleeping	47 (14.07)	135 (31.18)	14 (1.52)	22 (18.03)	2.06 (1.25–3.41)	0.09 (0.05–0.17)
Q3 > 1 h drowsy	242 (72.46)	315 (72.75)	20 (2.17)	28 (22.95)	8.96 (5.60–14.37)	0.01 (0.01–0.02)
Q4 Difficult to arouse	198 (59.28)	255 (58.89)	16 (1.74)	26 (21.31)	5.29 (3.30–8.49)	0.01 (0.01–0.02)
RBD						
Q5 Dream enact	24 (7.19)	115 (26.56)	12 (1.30)	26 (21.31)	1.34 (0.82–2.16)	0.17 (0.08–0.35)
Q6 Dream enact*	20 (5.99)	68 (15.70)	15 (1.63)	25 (20.49)	0.72 (0.43–1.20)	0.26 (0.13–0.51)
VH						
Q7 Eye tricks*	10 (2.99)	77 (17.78)	1 (0.11)	18 (14.75)	1.25 (0.72–2.18)	0.04 (0.01–0.28)
Q8 Sees things*	25 (7.49)	101 (23.33)	4 (0.43)	16 (13.11)	2.02 (1.14–3.57)	0.05 (0.02–0.16)
Q9 False visions	26 (7.78)	150 (34.64)	5 (0.54)	20 (16.39)	2.70 (1.61–4.54)	0.07 (0.03–0.17)
Q10 Sees things	28 (8.38)	157 (36.26)	7 (0.76)	26 (21.31)	2.10 (1.31–3.38)	0.08 (0.04–0.19)
Motor symptoms						
UPDRS 5-item score	1.59 ± 1.59	3.67 ± 3.27	0.34 ± 0.90	3.98 ± 3.24	0.97 (0.92–1.03)	0.45 (0.40–0.51)

Unless marked by the *, which distinguishes questions posed to the patient, the questions are posed to the patient’s caregiver

Abbreviations: *CF*, cognitive fluctuations; *RBD*, REM sleep behavior disorder; *VH*, visual hallucinations; *Q*, questions

### Comparison between results of the two questionnaires with the most recent DLB criteria (McKeith 2017)

Results of each questionnaire, LBCRS and AT-DLB, were compared with the final diagnosis made by each Center, based only on the most recent criteria. In every group considered (non-DLB, possible DLB, and probable DLB), the percentage of the agreement between the two assessments and the final diagnosis based on international diagnostic criteria was higher than 93%.

LBCRS and AT-DLB toolkits showed similar values of accuracy, sensitivity, and specificity, even though AT-DLB seemed to be less efficient (Table 6). On the contrary, when simultaneously considered in the logistic models to assess the AUC, AT-DLB was significantly more informative (Supplementary Materials) in assessing possible (0.13 ± 0.02 *p* < 0.001) and probable DLB (0.10 ± 0.01 *p* < 0.001).

**Table 6:** Analysis of sensitivity, specificity, accuracy, and AUC of the two toolkits vs. consensus criteria

	LBCRS vs. possible DLB	LBCRS vs. probable DLB	AT-DLB possible vs. possible DLB	AT-DLB probable vs. probable DLB
Accuracy	0.78	0.83	0.72	0.76
Sensitivity	0.53	0.85	0.13	0.75
Specificity	0.83	0.83	0.72	0.75
AUC	0.68 ± 0.02	0.84 ± 0.01	0.81 ± 0.01*	0.94 ± 0.008^

^*^Possible DLB: Comparison between LBCRS positive and AT-DLB possible

^Probable DLB: Comparison between LBCRS positive and AT-DLB probable

Abbreviations: *AUC*, area under curve

REM sleep behavior disorder was not correlated with possible DLB diagnosis, while it was directly correlated with probable DLB diagnosis.

## Discussion

Our results showed a prevalence of about 26% of possible or probable DLB diagnosis in the Dementia Centers as compared to the total diagnoses. With this second survey, the percentage of DLB diagnosis was comparable with the results of our first survey [4], higher than the prevalence reported in autopsy proven cohorts [1, 4]. Our data indicate a higher frequency of in vivo DLB diagnosis also compared to other European countries, according to recent observations [17, 18]. In a large UK’s multicenter study, DLB prevalence was 2.4–5.9% [17]. Despite a globally lower prevalence (3.4%), compared to our observation, a recent Belgian study found significant differences, in terms of DLB prevalence, according to the patients’ ethnicity [18]. DLB diagnosis was more frequent in North-Africans and Latin-American first-generation immigrants, compared to subjects born in Belgium [18]. Overall, these findings suggest a complex interplay between genetic and acquired factors that could underlie different epidemiological results for DLB. Furthermore, the high percentage of DLB diagnosis observed among the Italian Dementia Centers could be at least partially explained by the inclusion of cases of mixed dementia, which may have been classified as possible DLB [12, 13], as clinical diagnostic criteria for this entity are indeed very unspecific [1].

We found a good agreement between the two questionnaires, especially for probable DLB diagnosis. Even though the two questionnaires were validated before the most recent criteria were published [1], both toolkits have reached a high concordance with the current international diagnostic criteria. LBCRS toolkit showed a better internal consistency, as compared to AT-DLB, whereas the latter showed a better performance in identifying individuals with probable DLB (Table 6).

As regards as AT-DLB toolkit, CF was the most relevant factor, among all variables, for the accuracy of the diagnosis. CF are not an easy feature to be assessed in clinical practice. Clinician Assessment of Fluctuation (CAF) [19] is a helpful tool for the clinicians to identify properly this typical symptom of DLB. The AT-DLB toolkit could represent a suitable alternative for CF assessment. Indeed, positive answer to the CF item of AT-DLB correlated with a higher risk of DLB diagnosis, only followed by VH items. As regards to LBCRS toolkit, and in accordance with literature data, which reports that VH are the most specific symptom in differentiating DLB from AD [20], VH were the most relevant symptom, followed by RBD, for the accuracy of diagnosis.

From a speculative point of view, the combination of the two toolkits could lead to a superior diagnostic accuracy for the evaluation of CF, VH, and RBD, which are currently considered as core clinical features.

Limitations of this study are related to missing information during data collection, such as comorbidities and the final clinical diagnosis made for each patient recruited in each Center. This issue did not allow to estimate the presence of other dementias which could have been diagnosed as DLB (especially possible DLB). A further limitation is the low number of patients whose diagnose was corroborated through the study of biomarkers. Only a very low percentage of patients were studied by indicative or supportive biomarkers including DaT-SPECT, myocardial scintigraphy, FDG-PET, quantitative EEG, polysomnography.

To conclude, standardization in the clinical assessment of DLB symptoms should be regarded as a priority, until the discovery of novel and optimal biomarkers.

## Supplementary Information

Below is the link to the electronic supplementary material.

Supplementary file1 (DOCX 248 KB)
