# Supplementary material for: Accuracy of the clinical diagnosis of dementia with Lewy bodies (DLB) among the Italian Dementia Centers: a study by the Italian DLB study group (DLB-SINdem)
Source: Neurol Sci. 2022 Mar 4;43(7):4221–9. doi: 10.1007/s10072-022-05987-z (PMC9213280; doi:10.1007/s10072-022-05987-z)
Supplement: Supplementary file 1 — Supplementary file1 (DOCX 248 KB) [file 10072_2022_5987_MOESM1_ESM.docx]

**Supplementary Materials**

Supplementary Figure 1. Receiver Operating Characteristics (ROC) based on Logistic Regression Models for Probable and Possible DLB diagnoses.


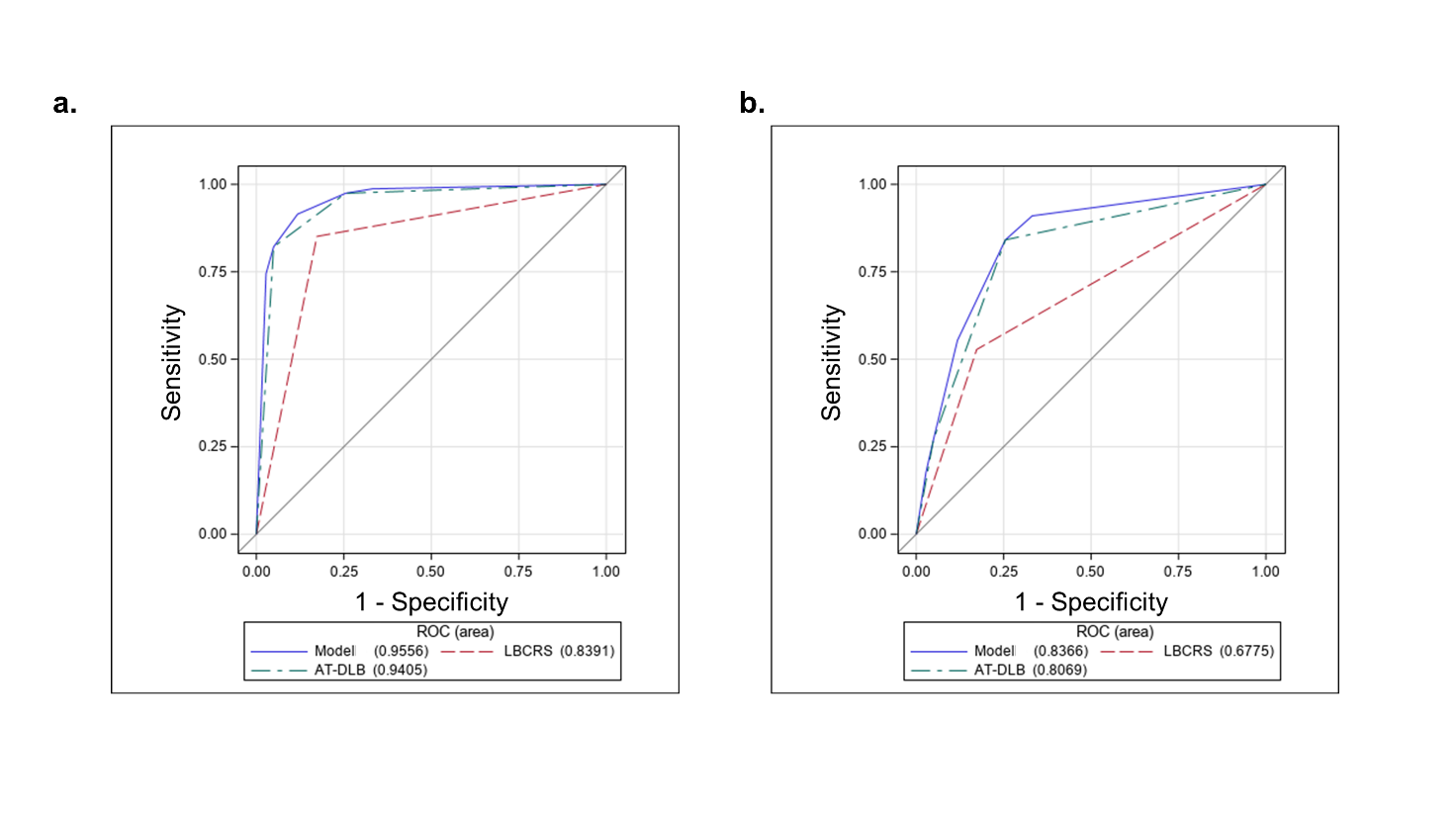


Receiver Operating Characteristics (ROC) for the estimation of the Area Under the Curve (AUC), with DLB criteria as the dependent variable and the LBCRS and AT-DLB toolkits as the independent variables, simultaneously considered. **Panel a** shows the ROC for the “Probable DLB” diagnosis, where those classified as “Possible DLB” were excluded from the analysis. **Panel b** depicts the opposite situation (ROC for “Possible DLB” diagnosis, with the exclusion of subjects with Probable DLB).
